# Albumin uptake in human podocytes: a possible role for the cubilin-amnionless (CUBAM) complex

**DOI:** 10.1038/s41598-017-13789-z

**Published:** 2017-10-20

**Authors:** Lisa Gianesello, Giovanna Priante, Monica Ceol, Claudia M. Radu, Moin A. Saleem, Paolo Simioni, Liliana Terrin, Franca Anglani, Dorella Del Prete

**Affiliations:** 1 Clinical Nephrology, Department of Medicine - DIMED, University of Padua, 35129 Padua, Italy; 2 Thrombotic and Hemorrhagic Diseases Unit, Department of Medicine - DIMED, University of Padua, 35129 Padua, Italy; 3 Academic and Children’s Renal Unit, Dorothy Hodgkin Building, BS8 1TH Bristol, United Kingdom

## Abstract

Albumin re-uptake is a receptor-mediated pathway located in renal proximal tubuli. There is increasing evidence of glomerular protein handling by podocytes, but little is known about the mechanism behind this process. In this study, we found that human podocytes *in vitro* are committed to internalizing albumin through a receptor-mediated mechanism even after exposure to low doses of albumin. We show that these cells express cubilin, megalin, ClC-5, amnionless and Dab2, which are partners in the tubular machinery. Exposing human podocytes to albumin overload prompted an increase in *CUBILIN*, *AMNIONLESS* and *CLCN5* gene expression. Inhibiting cubilin led to a reduction in albumin uptake, highlighting its importance in this mechanism. We demonstrated that human podocytes are committed to performing endocytosis *via* a receptor-mediated mechanism even in the presence of low doses of albumin. We also disclosed that protein overload first acts on the expression of the cubilin-amnionless (CUBAM) complex in these cells, then involves the ClC-5 channel, providing the first evidence for a possible role of the CUBAM complex in albumin endocytosis in human podocytes.

## Introduction

Loss of proteins is the hallmark of tubular and glomerular diseases, and may be due to structural and/or functional alterations involving different cell types^1^. Albuminuria is a powerful independent predictor of renal disease progression, cardiovascular disease, and death in patients with renal disease, hypertension, diabetes, and vascular disease, and in the general population too^1,2^.

The most widely studied endocytosis mechanism is receptor-mediated endocytosis. This process involves the cooperation between soluble molecules in the extracellular fluid and cell-surface receptors and the subsequent internalization of these complexes *via* clathrin-coated vesicles that originate from the cellular membrane^3,4^. This mechanism is considered one of the key features of proximal tubular epithelial cells (PTECs). Protein endocytosis at tubular level relies on an active receptor-mediated pathway that mainly involves megalin (*LRP2*), cubilin (*CUBN*), amnionless (*AMN*), disabled-2 (*DAB2*) and ClC-5 (*CLCN5*)^1^. Megalin and cubilin are particularly important in this process. They bind several ligands, fulfilling their task in receptor-mediated endocytosis by mediating the delivery of soluble molecules to the lysosomes of PTECs, and being recycled in the process^5,6^. While various proteins (such as retinol binding protein) apparently bind exclusively to megalin, others (such as vitamin D binding protein) bind with similar affinity to both receptors. Albumin is thought to have greater binding affinity for cubilin rather than megalin^7^.

The CUBAM complex is formed by AMN and its well-known partner Cubilin. This complex is localized on the plasma membrane in order to play its functional role and it was already reported its independence from megalin activity. Cubilin is tightly connected with AMN for its normal translocation from the endoplasmic reticulum to the membrane, and for the consequent endocytosis process^8,9^. The CUBAM complex is implicated in the endocytosis of various substances in both the intestine and the kidneys. In proximal tubular cells, cubilin and megalin are required to clear the ultrafiltrate by filtered molecules, in order to obtain almost protein-free urine. Albumin was one of the first ligands to be identified, focusing on these receptors for its excretion^10,11^.

Like renal PTECs, podocytes have been found capable of internalizing albumin. Several Authors described and quantified an albumin endocytosis function in murine and rat podocytes, both *in vitro* and *in vivo* ^12–14^. Recently, it has been demonstrated that human podocytes can endocytose proteins such as albumin using kinetics consistent with a receptor-mediated process when placed in a medium with high doses of albumin^15,16^.

In the last few years, it has been demonstrated that human podocytes express megalin and cubilin both *in vivo* and *in vitro* ^17,18^. Our own group found ClC-5 expressed in the glomeruli of normal and proteinuric human kidneys, and particularly in podocytes^19^. In the same study, ClC-5 was overexpressed at both mRNA and protein level in the glomeruli of biopsies obtained from patients with diabetic nephropathy and membranous glomerulonephritis.

How human podocytes perform endocytosis is still unclear. The aim of the present study was therefore to clarify whether components of the typical tubular protein uptake system - such as megalin, cubilin, ClC-5, AMN and Dab2 - are involved in the mechanism underlying albumin internalization in human podocytes *in vitro*, and how protein overload might affect the expression of this system.

## Results

### Human podocytes internalize low doses of albumin via a receptor-mediated process

**Figure 1:**
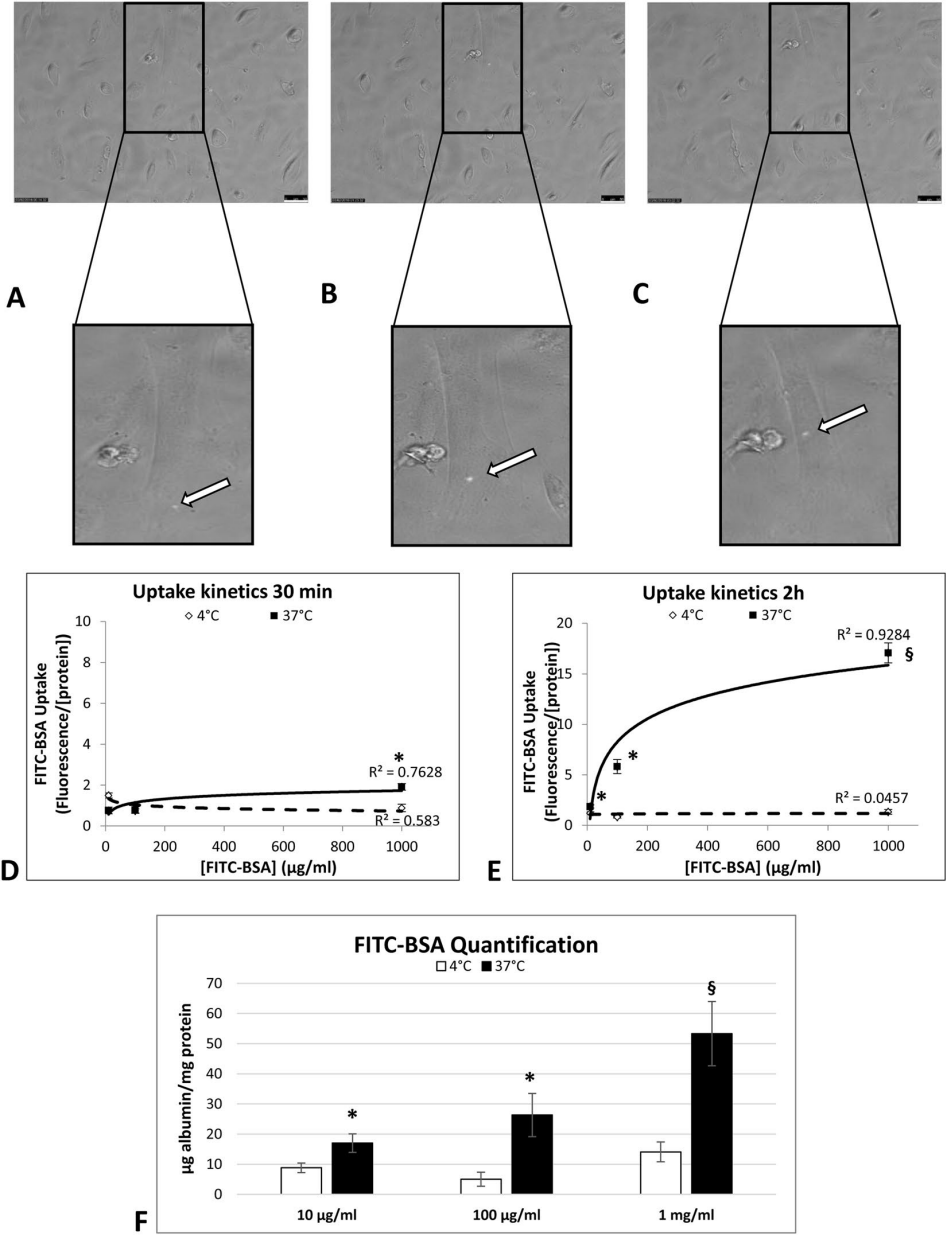
Podocytes perform receptor-mediated uptake of albumin even at low doses. Time lapse experiments disclosed the ability of podocytes to internalize albumin (FITC-BSA 10 µg/ml). Representative images show vesicles containing albumin (green) at different time points: 5 h 30 min (**A**), 6 h 39 min (**B**), 8 h 18 min (**C**). The white arrow follows the movement of one vesicle from the periphery of the cell to the perinuclear region. Objective 20X/0.4. Scale bar 50 µm. Albumin uptake follows receptor-mediated kinetics in human podocytes. At 30 minutes, only the 1 mg/ml concentration was significant (**D**); after 2 hours, FITC-BSA was significantly internalized at all doses tested by comparison with non-stimulated control cells (**E**). Albumin uptake increased until saturation at 37 °C (continuous line), with abolition of the process at 4 °C (dotted line). Graph F shows significant quantity of FITC-BSA internalized by podocytes at 37 °C comparing to 4 °C. Data are shown as the average ± SD of three different experiments in triplicate. *P < 0.05; ^§^P < 0.01.

In order to track albumin uptake, we monitored conditionally immortalized human podocytes overnight after stimulation with a low dose (10 µg/ml) of fluorescent albumin (FITC-BSA). Starting from 2 hours later, we confirmed the ability of podocytes to internalize albumin. The signal was detected as a green vesicle moving from the cellular membrane to the perinuclear region (Fig. 1A–C), and then disappearing. This process started between 2 and 15 hours after stimulation, and involved 23 ± 9% of the cells observed.

An endocytosis and binding assay was performed to establish the kinetic parameters of albumin uptake in human podocytes. The podocytes were incubated with increasing concentrations of FITC-BSA at 37 °C (specific uptake) or at 4 °C (non-specific uptake). Fluorescence intensity was recorded 30 minutes and 2 hours after stimulation at both temperatures to see which mechanism underlies the uptake process. Albumin internalization occurred after 2 hours of stimulation, confirming the results obtained in our time lapse experiments. The exponential profile obtained at 37 °C, but not at 4 °C, ruled out any non-specific binding of the molecule and confirmed the hypothesis of a receptor-mediated mechanism (Fig. 1D and E). FITC-BSA quantification indicated that the uptake process at 37 °C followed a significantly dose-dependent trend (10 μg/ml: 16.99 ± 3.06 µg albumin/mg protein, 100 μg/ml: 26.31 ± 7.18 µg albumin/mg protein, and 1 mg/ml: 53.31 ± 10.68 µg albumin/mg protein) by comparison with the process at 4 °C (10 μg/ml and 100 μg/ml P < 0.05; 1 mg/ml P < 0.01) (Fig. 1F).

### Human podocytes express the CUBAM complex which colocalizes with albumin

**Figure 2:**
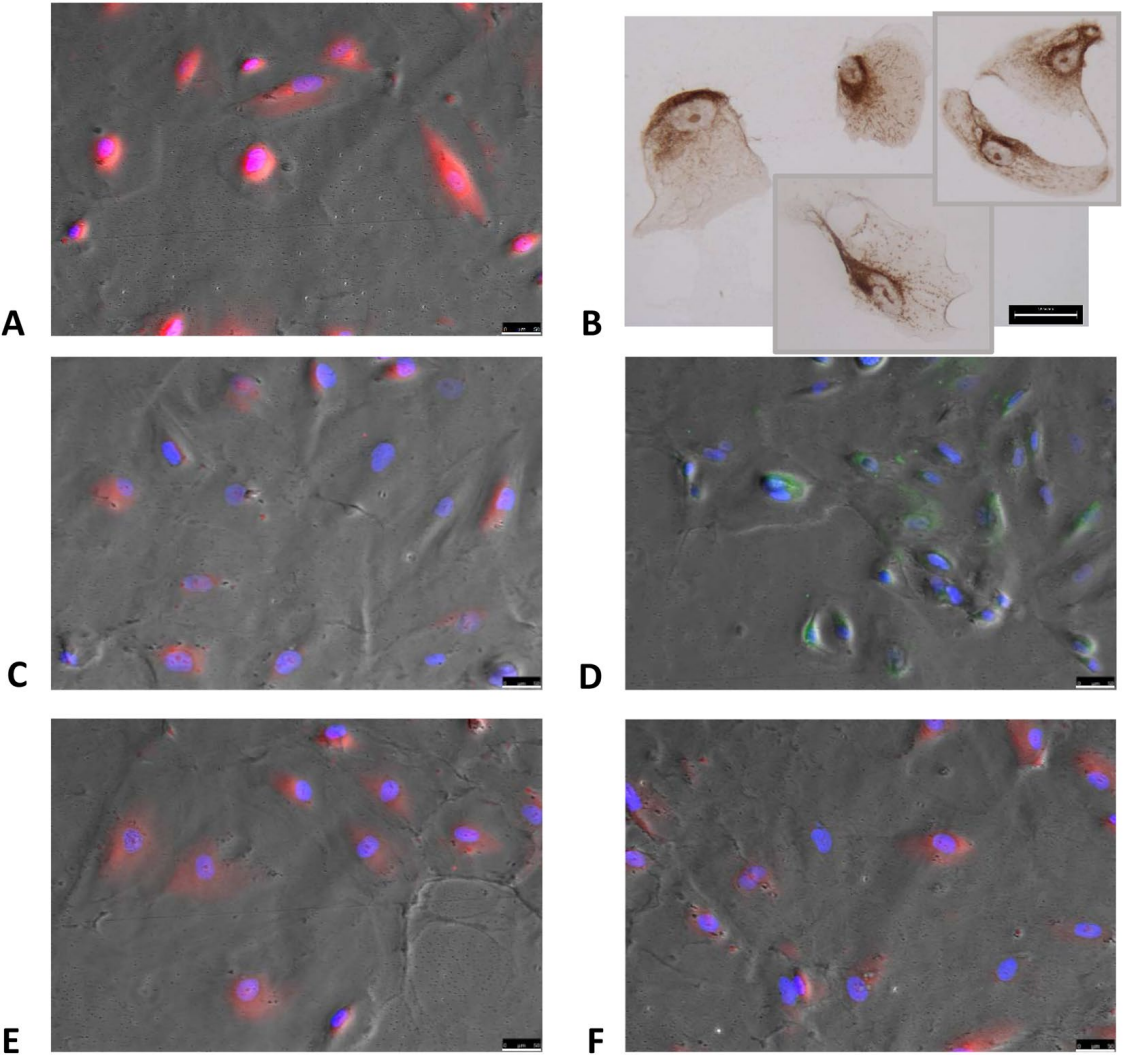
Podocytes express the typical tubular uptake machinery. Immunostaining of human podocytes disclosing: (**A**) podocin (red), (**B**) ClC-5 (brown), (**C**) megalin (red), (**D**) cubilin (green), (**E**) Dab2 (red), (**F**) AMN (red). Immunohistochemistry objective 20X/0.45, immunofluorescence objective 20X/0.4. Scale bar 50 µm.

Since our results indicated that a receptor-mediated mechanism was involved in albumin internalization in human podocytes, we investigated the presence of megalin, cubilin, Dab2, AMN and ClC-5 (normally present in PTECs) in human podocytes *in vitro* under standard conditions. We confirmed the presence of megalin and cubilin, and newly identified the presence of ClC-5, Dab2 and AMN (Fig. 2B–F).

**Figure 3:**
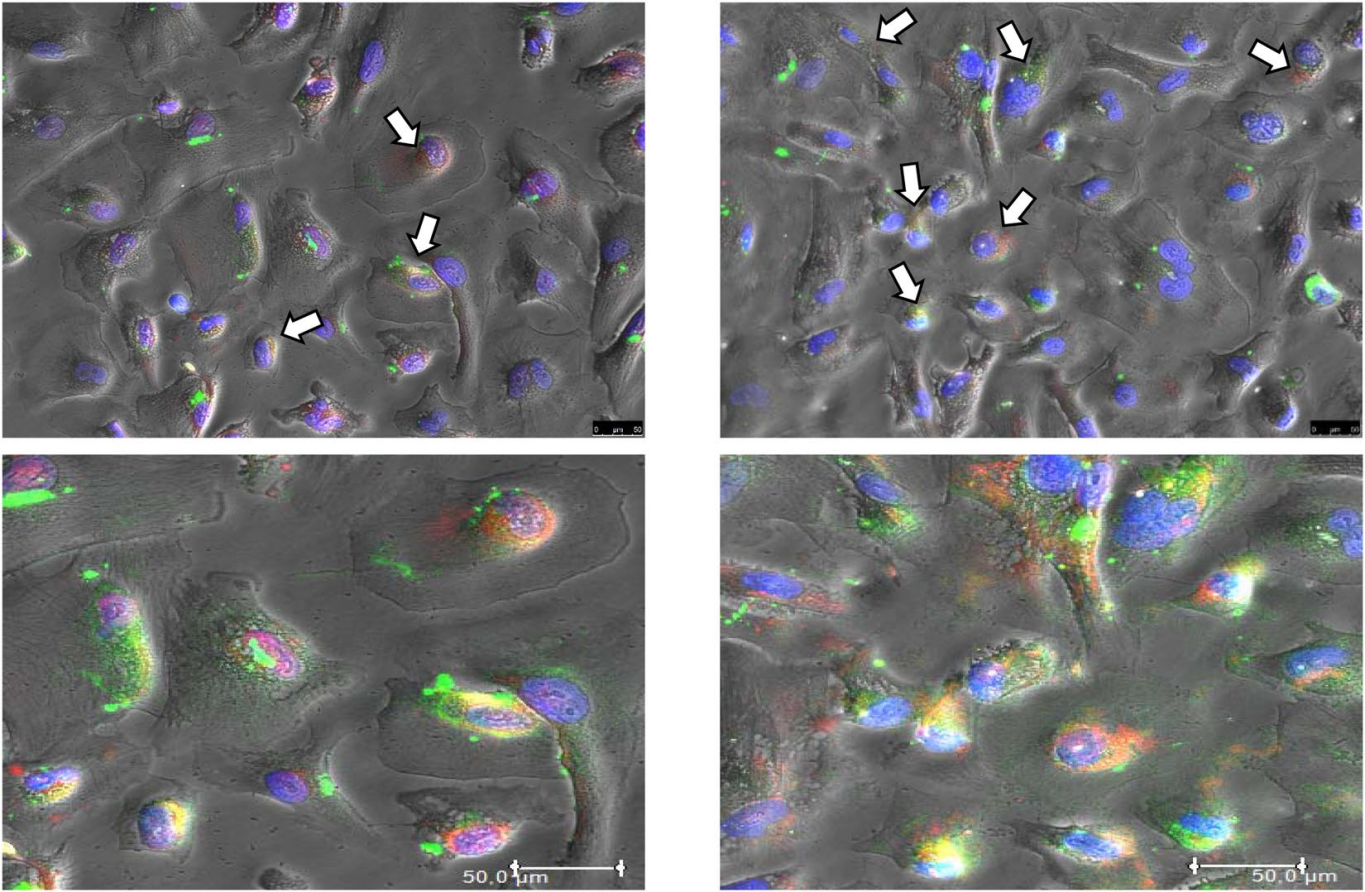
Albumin colocalizes with megalin and cubilin in human podocytes. Immunolabeling shows colocalization (in yellow/orange) of megalin (left) and cubilin (right) with albumin after 24 hours of incubation. Lower panel are zoom-in of respectively upper images. Statistical analysis using Pearson’s coefficient (*Rr*) with LAS-AF software disclosed a significant correlation between cubilin and FITC-BSA (*Rr* = 0.71), and a non-significant correlation between megalin and FITC-BSA (*Rr* = 0.45). Green: FITC-BSA; Red: megalin (left) or cubilin (right); White arrows indicate colocalization. Blue: DAPI. Objective 20X/0.4. Scale bar 50 µm.

After 2, 4, 8 and 24 hours of incubation with FITC-BSA 10 µg/ml, podocytes were immunostained to examine the colocalization of fluorescent albumin and megalin or cubilin. The highest colocalization signal was apparent 24 hours after stimulation (Fig. 3). The signal was mainly perinuclear, confirming the movement of BSA vesicles observed in the time lapse experiments (Fig. 1A–C). A quantitative colocalization analysis was run to estimate the degree of colocalization 24 hours after stimulation using Pearson’s correlation coefficient (*Rr*) on at least 150 positive cells, as described elsewhere^20^. Although a colocalization was present for both receptors, only the correlation between cubilin and FITC-BSA was statistically significant (*Rr* = 0.71), while the correlation between megalin and FITC-BSA lacked statistical significance (*Rr* = 0.45).

### Albumin modulates components of the tubular protein uptake machinery in human podocytes

To rule out the detrimental effect of protein overload on podocytes, we estimated the percentage of dead cells at 24, 48 and 72 hours after incubation with BSA (range 10 µg/ml–30 mg/ml). We found no significant increase in necrosis and/or apoptotic events at any of the times and concentrations tested (Supplementary Figure S1A). Nor was there any apparent effect on the podocyte-specific marker podocin after BSA treatment, indicating that protein overload did not affect cellular differentiation (Supplementary Figure S1B).

**Figure 4:**
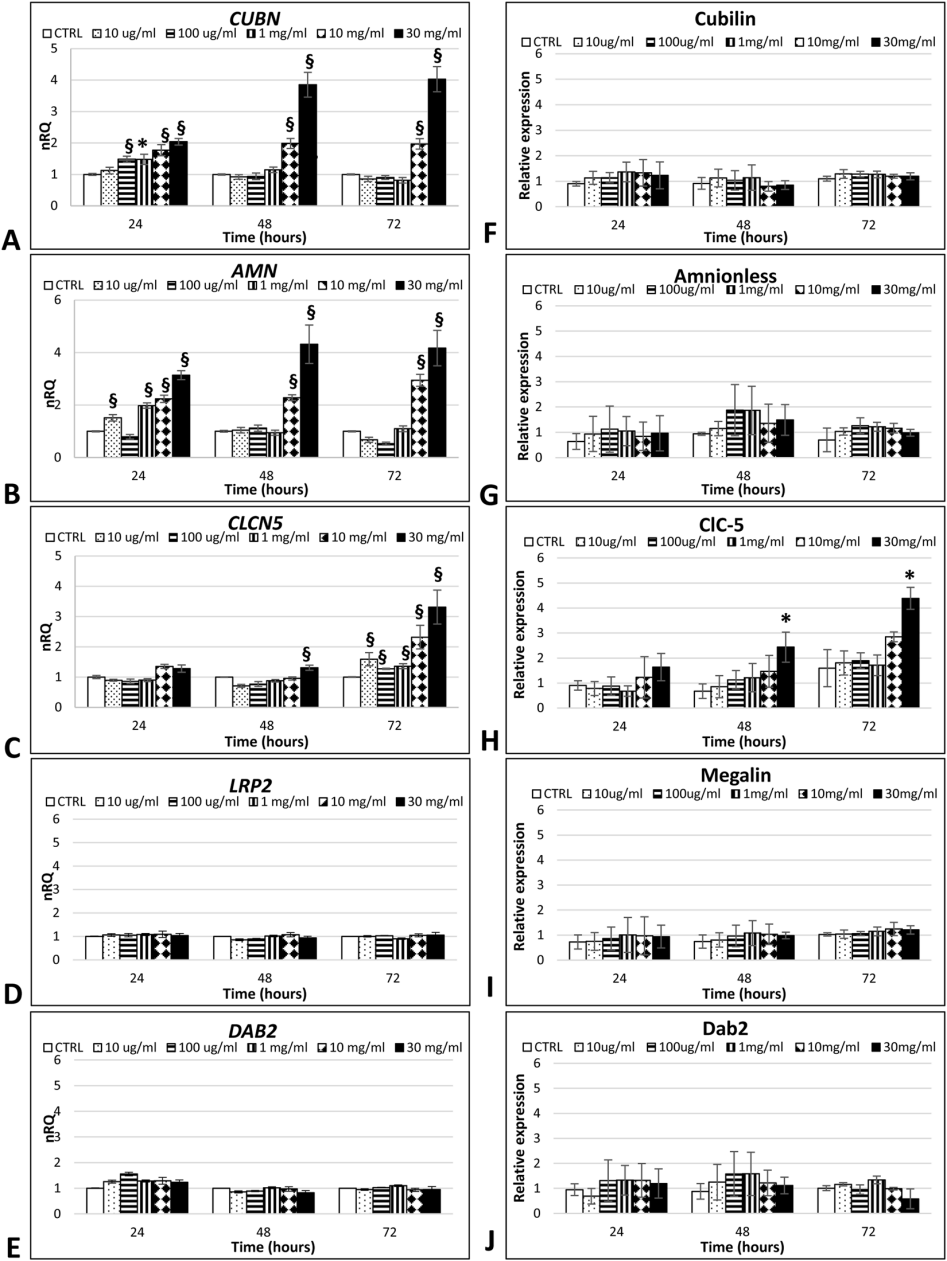
Protein overload modulates the expression of the CUBAM complex. Gene expression analysis of the macromolecular system showed an increase in *CUBN* (**A**), *AMN* (**B**)and *CLCN5* (**C**) genes. Data are given as the geometrical average ± SE of three independent experiments in duplicate. nRQ: normalized Relative Quantity. Protein expression analysis of the macromolecular system only showed an increase in ClC-5 (**H**) expression at the longest time and highest dose considered. Data are given as the average ± SD of four different experiments. CTRL: control cells, *P < 0.05, ^§^P < 0.01.

In agreement with immunostaining results, gene expression analysis confirmed the presence of *LRP2*, *CUBN*, *CLCN5*, *AMN* and *DAB2* in standard conditions. Incubating podocytes with increasing concentrations of albumin, we found a significant upregulation in *CUBN* and *AMN* expression, especially at the highest BSA concentrations (10 and 30 mg/ml) (P < 0.01), which did not give rise to any increase in protein levels (Fig. 4A,B and F,G). ClC-5 gene and protein expression were significantly increased (P < 0.01 and P < 0.05 respectively) after chronic (48–72 hours) protein overload (10 and 30 mg/ml) (Fig. 4C and H). BSA exposure did not modulate Dab2 or megalin, at either mRNA or protein levels (Fig. 4D,E and I,J).

### Cubilin mediates albumin endocytosis in human podocytes

**Figure 5:**
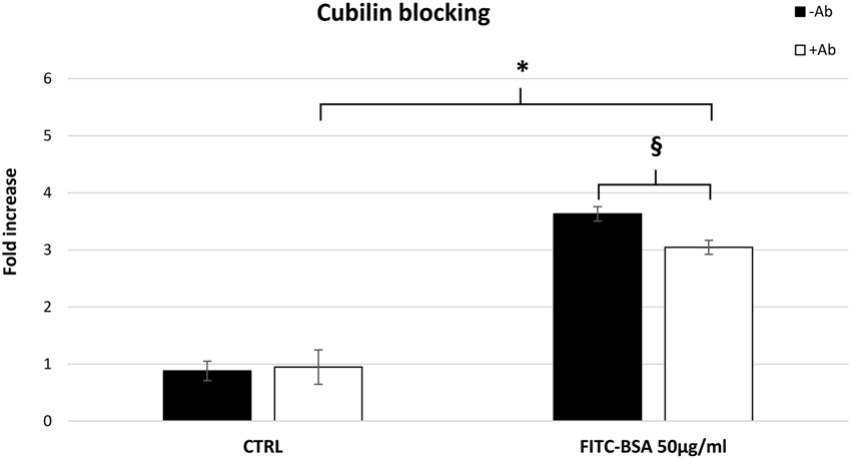
Cubilin is involved in albumin uptake. Antibody-mediated inhibition of cubilin (white blocks) led to a significant decrease in albumin endocytosis, indicating an involvement of this receptor in the protein uptake process in mature podocytes. Data are shown as the average ± SD of two different experiments in triplicate. CTRL: control cells. *P < 0.05, ^§^P < 0.01.

In order to establish whether cubilin was directly involved in albumin internalization, we inhibited the albumin binding sites on cubilin and quantified FITC-BSA internalization 2 hours after stimulation. As shown in Fig. 5, we found a significant inhibition of albumin internalization in human podocytes after immunological blocking of cubilin (P < 0.01), indicating that cubilin was involved in the uptake mechanism. However, the lack of total endocytosis process block in the presence of anti-cubilin antibody, suggests that some albumin endocytosis occurs through alternate pathways, and that the cubilin-facilitated path is not the exclusive endocytic pathway involved in albumin internalization by human podocytes.

## Discussion

In the present study we deep insight into albumin endocytosis in human podocytes adding new information about this mechanism. Several Authors had already demonstrated albumin uptake in cultured podocytes but using doses of albumin at least a hundred times higher (range 1.5–3 mg/ml)^12,21^. This is the first time the number of cells performing albumin uptake at low doses has been quantified. Our time lapse microscope strategy prevented us from using high doses of albumin, but enabled us to disclose the capacity of human podocytes to internalize albumin even at low doses. This albumin concentration can be considered as normal condition^22^. While previous studies were conducted in protein overload media^12–14^, for the first time we demonstrated that human podocytes were able to internalize albumin under normal condition, suggesting that these cells are committed to protein uptake.

Endocytosis is a well-known process that can occur in two different ways: one involves the non-specific binding of the molecule to the cell membrane and its subsequent internalization; the other is receptor-mediated endocytosis, which is characterized by typical saturation kinetics^23^. In proximal tubular cells, where the protein uptake machinery is typically expressed, it takes about 30 minutes for the process of endocytosis to take place (range of albumin 100 µg/ml-1 mg/ml)^24^. In our low-dose (FITC-BSA 10 μg/ml) experiments on human podocytes, this process needed at least 2 hours to occur. While previously-reported data were obtained in murine podocytes or human urine-derived podocyte-like cells (HUPEC), and using higher concentrations of albumin^12,15^, our results are the first to demonstrate the receptor-mediated endocytosis process even at very low doses of albumin.

Since our results indicated that a receptor-mediated mechanism was involved in albumin internalization in human podocytes, we investigated the presence of megalin, cubilin, Dab2, AMN and ClC-5 in human podocytes *in vitro* under standard conditions. Our results extend current knowledge on the components of this macromolecular system typically expressed in PTECs, demonstrating the expression not only of megalin and cubilin^17,18^, but also of ClC-5, AMN and Dab2 in human podocytes *in vitro*. Albumin internalization can be mediated by megalin or cubilin, or both, so we investigated whether these receptors were involved in albumin binding. We demonstrated a significant colocalization between FITC-BSA and cubilin, indicating a possible role for this receptor in albumin internalization by human podocytes *in vitro*.

The presence of components of the protein uptake machinery in mature human podocytes led us to investigate whether protein overload could affect the level of expression of this system. Prolonged exposure (96 hours) to albumin has been found deleterious to murine podocytes^25^, and exposing HUPEC or murine podocytes to high concentrations of albumin (range 5–10 mg/ml) for 24 and 48 hours reportedly increased cell death^26,27^. Our results differ from those obtained by Yoshida *et al*., possibly due to differences in our experimental design as regards albumin doses, timing of stimulation, and cell type^27^. Comparing our data with those obtained by Okamura *et al*. (who used similar doses of albumin) showed that human podocytes internalize less albumin than HUPEC^26^. But should human podocytes and HUPEC be considered as the same cell type? HUPEC express several podocytes markers (WT-1, synaptopodin and nestin), but they lack important features of fully-differentiated podocytes (including nephrin and podocin), when cultured under standard conditions at least^26^.

Our group had previously demonstrated ClC-5 overexpression in the glomeruli of proteinuric patients, at both mRNA and protein level, suggesting a link between this channel and proteinuria^19^. The gene expression data obtained in this study support our previous hypothesis. We demonstrated that protein overload in human podocytes first takes effect on the expression of the CUBAM complex, then involves the ClC-5 channel in the endocytic vesicle acidification. The absence of a correspondence between cubilin mRNA and protein expression was already described^28^ and may be due to post-transcriptional events, as reported for other molecules^29^. To our knowledge, no data are available on the expression profile of this system at glomerular level in the protein overload setting. Liu *et al*. reported that albumin overload in PTECs induced a slight increase in megalin and cubilin protein expression at 24 hours, with a marked decrease at 48 hours. The decline was associated with the presence of albumin-induced apoptosis^30^. In human podocytes, we found no such decline in either cubilin or megalin mRNAs and proteins, probably due to the absence of any increase in cell death.

Given the absence of any modulation of megalin expression at mRNA or protein level, and the presence of a significant correlation between cubilin and FITC-BSA in colocalization experiments, we focused our attention on the CUBAM complex. Incubating podocytes with an anti-cubilin antibody, we observed a significant but partial inhibition of the albumin uptake process. This incomplete blocking may be due to several reasons. First, the cubilin-facilitated pathway may not be the only endocytotic mechanism of albumin uptake in podocytes. We cannot exclude a role for megalin since we confirmed its presence in human podocytes *in vitro* as previously demonstrated by the Christensen’s group *in vitro* and *in vivo* ^17^. Moreover, a colocalization between FITC-BSA vesicles and the neonatal Fc receptor (FcRn) in HUPEC has already been reported^15^. These data suggest a collaboration between cubilin and megalin and/or between cubilin and FcRn in human podocytes. Second, the incomplete blocking may be a consequence of internalization of the complex antibody-CUBAM as seen previously for megalin in canine tubular cells^31^. Further studies will be needed to shed more light on the involvement of other molecules in albumin endocytosis by cubilin in human podocytes.

Podocytopathies are the most common group of glomerular disorders leading to proteinuria. The knowledge of the mechanisms involved in albumin handling in podocytes is a fundamental element not only to understand pathogenesis of glomerulopathies but also to identify novel drug targets. From this perspective, receptor-mediated endocytosis is an important mechanism involved in the physiology of several cell types, and numerous proteins and signal transduction molecules are needed to guarantee this function. Albumin endocytosis by proximal tubules is a well-known phenomenon, involving the macromolecular complex consisting of megalin, cubilin, AMN, Dab2 and ClC-5. Various studies have demonstrated albumin uptake in podocytes. What remains to be clarified is the mechanism underlying this process. For the first time, we demonstrated that human podocytes are committed to performing endocytosis *via* a receptor-mediated mechanism even in the presence of low doses of albumin, thus supporting their ability to do so. We identified the components of the typical tubular protein uptake system - such as megalin, cubilin, ClC-5, AMN and Dab2 - in human podocytes *in vitro* too. We demonstrated that protein overload first acts on the expression of the CUBAM complex in these cells, then involves the ClC-5 channel in the endocytic vesicle acidification. Although human podocytes express the components of the tubular uptake machinery, they seem to perform the receptor-mediated endocytosis of albumin in different ways from PTECs. In fact, albumin uptake in PTECs requires coordinated collaboration between megalin and cubilin, while cubilin was found to probably have a more important role than megalin in human podocytes. Our findings provide the first evidence for a possible role of the CUBAM complex in albumin endocytosis in human podocytes *in vitro*.

## Methods

### Human podocytes

Human podocytes were kindly provided by Prof. Saleem and maintained in RPMI 1640 medium supplemented with 10% fetal bovine serum (FBS; Sigma-Aldrich), Insulin-Transferrin-Selenium supplement (ITS; Sigma-Aldrich), 2 mM L-glutamine and antibiotic mixture, as previously reported^32^. To stimulate cell proliferation, podocytes were cultivated at 33 °C in 5% CO_2_ (permissive conditions). To induce differentiation, they were maintained at 37 °C in 5% CO_2_ (non-permissive conditions) for at least 2 weeks. The end of the differentiation process was assessed on the grounds of podocin positivity a well-known component of the slit diaphragm (Fig. 2A). Cell density was kept below 90% to allow differentiation. Cells were used between passages 7 and 12. To albumin effects, cells were cultured in serum deprivation (1%) starting from 24 hours before stimulation.

### Time lapse fluorescence imaging

To monitor BSA endocytosis in real time, podocytes were seeded on 24-well plates, then incubated overnight with FITC-BSA (Albumin Fluorescein isothiocyanate conjugate from bovine (A9771) Sigma Aldrich) 10 µg/ml in medium without phenol red. Images were acquired every 3 minutes. At least 15 cells for each acquisition were analyzed to identify the presence of BSA vesicles. Time lapse images were obtained using Leica Application Suite (LAS-AF) 3.1.1 software (Leica Microsystems). All acquisitions were performed with an inverted fluorescence microscope (DMI600CS-TCS SP8, Leica Microsystems) placed within a home-made, temperature-controlled enclosure set at 37 °C and 5% CO_2_ for live cell imaging. Experiments were run twice and acquired with 20X/0.4 objective using a DFC365FX camera (Leica Microsystems).

### Endocytotic uptake analysis

Endocytosis was quantified as described by Coffey *et al*.^24^ with some modifications. Briefly, mature podocytes seeded on 6-well plates were incubated with increasing concentrations of FITC-BSA (10, 100 and 1000 µg/ml) for 30 minutes and 2 hours at both 37 °C and 4 °C. Intracellular fluorescence was measured in a single-beam fluorimeter (Victor X3, PerkinElmer) at an excitation wavelength of 490 ± 10 nm and emission wavelength of 520 ± 10 nm. Fluorescence was normalized for the amount of cells as the quantity of protein. Cell lysates were obtained by means of a physical procedure (freezing in liquid nitrogen and defrosting in water at 37 °C) in lysis buffer containing 1 mM PMSF. Proteins were quantified at 280 nm with a Nanodrop ND-100 spectrophotometer (Celbio). Results were expressed as fluorescence/milligram of protein.

### Immunofluorescence (IF)

For the immunofluorescence (IF) analysis, mature podocytes cultivated in standard conditions were seeded on 8-well chamber slides and fixed with cold methanol for 5 minutes at room temperature (RT). For colocalization experiments, mature podocytes on 96-well plates were changed to 1% serum medium and left overnight. Then the cells were incubated with FITC-BSA (10 µg/ml) for 2, 4, 8 and 24 h in the same medium. Finally, cells were fixed with cold methanol for 5 minutes at RT. Cells were treated as previously described^33^ blocking with 10% normal donkey serum (Abcam) for 30 min at RT. Samples were incubated with primary antibody (Supplementary Table S1) diluted in BSA 5% in PBS at 4 °C overnight. Cells were incubated with the appropriate fluorescent secondary antibody diluted in BSA 5% in PBS for 1 h at RT (Supplementary Table S1), and nuclei were counterstained with DAPI diluted 1:1,000 in PBS for 5 min at RT. Negative controls were run by omitting the primary antibody. Images were acquired using a DMI6000CS-TCS SP8 fluorescence microscope and analyzed using the LAS-AF software. Experiments were done in duplicate and acquired with 20X/0.4 objective.

### Immunohistochemistry (IHC)

Mature podocytes were cultivated on 8-well chamber slides in standard conditions and fixed with cold methanol for 5 minutes at RT. Specimens were treated as previously described^19^. Cells were incubated overnight with a rabbit antibody against ClC-5 (Sigma-Aldrich, Supplementary Table S1) diluted in PBS at 4 °C in a humidified chamber. The specificity of the immunolabeling was confirmed by incubation without primary antibody or with nonimmune rabbit IgG (Sigma-Aldrich). Images were acquired using the Diaplan light microscope (Leitz). Experiments were run twice and acquired with 20X/0.45 objective using a Micropublisher 5.0 RTV camera (Q Imaging).

### Apoptosis assay

Annexin V FITC staining was assessed with a commercial kit paired with propidium iodide (PI) (Affymetrix-eBioscience) following the manufacturer’s instructions. Briefly, cells treated in T-75 flasks were detached with trypsin 0.05% after 24, 48 and 72 hours of BSA stimulation, and washed in PBS. After resuspending mature podocytes in binding buffer, 200,000–500,000 cells/ml were marked with Annexin V-FITC for 10 minutes at RT. The cells were then washed with binding buffer and resuspended in the same buffer with PI. Apoptotic stages were assessed by flow cytometry using a CytoFLEX cytometer (Beckman Coulter), and analyzing 10,000 cells for each condition. Cells without Annexin V and PI were used as negative control. Images were analyzed using the CytExpert software (Beckman Coulter).

### RNA extraction and cDNA synthesis

Podocytes were cultured in T-75 flasks and incubated with increasing concentrations of albumin (BSA; Sigma-Aldrich) (range 10 µg/ml–30 mg/ml) for 24, 48 and 72 hours. Control cells were cultured without stimulation. The RNeasy Mini Kit (Qiagen) was used to isolate total RNA from the cells. The RNA was quantified with the Nanodrop ND-100 spectrophotometer. RNA purity was checked from the A260/A280 ratio and its integrity was tested by capillary electrophoresis on the Agilent RNA Nano chip with the Agilent 2100 Bioanalyzer (Agilent Technology). Only RNA with an RNA integrity number of at least of 9 was used for Real-Time PCR analyses. RNA was retro-transcribed from a starting quantity of 100 ng in a final volume of 20 µl. The reaction mix was prepared as follows: 5 mM MgCl_2_; 1 mM dNTPs; 2.5 µM random hexamers; 1 U RNase inhibitor; 2.5 U MuLV reverse transcriptase (ThermoFisher Scientific) in 50 mM KCl, 10 mM Tris HCl pH 8.3. Reactions were performed on the 2720 thermal cycler (ThermoFisher Scientific) applying the following thermal profile: RT for 10 min, 42 °C for 30 min, 65 °C for 5 min, 4 °C for 5 min.

### Real Time PCR

Primer pairs for the region of interest were designed according to stringent parameters to ensure successful assays and a convenient experimental design by using Primer3 software ver. 4.0 (http://primer3.ut.ee). The NCBI Primer-BLAST program was used for in silico specificity analysis (www.ncbi.nlm.nih.gov/tools/primer-blast/index.cgi). Microchip electrophoresis on the Agilent 2100 BioAnalyzer, Sanger sequencing, and melting curve analysis were used to check the specificity of the PCRs. Amplification curves were established for all primers and resulted in efficiencies of at least 85%. Primers used are listed in Supplementary Table S2. For each fragment to analyze, 1 µl of cDNA was amplified in a 20 µl final volume of reaction mix using SYBR Green Master Mix (EurX) according to the manufacturer’s instructions. Reactions were performed on the RotorGene (Corbett Research). Appropriate primer dilutions and annealing temperatures are given in Supplementary Table S3. Data were analyzed using the ΔΔCt method, normalizing on three different housekeeping genes (*GAPDH*, *HPRT1*, *β2-MICROGLOBULIN*) according to the MIQE guidelines^34^.

### In-Cell Western (ICW) assay

Protein expression was assessed in mature podocytes cultivated on 96-well plates fixed with cold methanol for 10 minutes at RT after stimulation. Cells were washed five times with 0.1% Triton X-100 in PBS, then blocked in a blocking solution of 5% milk in 0.1% Triton X-100 in PBS for 40 minutes at RT with moderate shaking. Samples were incubated with primary antibody (Supplementary Table S1) diluted in the same medium at 4 °C overnight in a humidified chamber. Then, five washes with 0.1% Triton-X 100 in PBS washing solution were carried out. Secondary antibodies were diluted in blocking solution, as reported in Supplementary Table S1, and incubated for 1 hour at RT with gentle shaking. The intensity of the labelled proteins was measured using the Odyssey CLx imaging system (LI-COR). Negative controls were run by omitting primary antibody. Background values were obtained by omitting primary and secondary antibodies. Each experiment was run in quadruplicate. Signals were normalized for the amount of cells measured by methylene blue staining as described elsewhere^35^. Briefly, cells were stained for 30 min with 1% methyl blue in 0.01 M borate buffer, pH 8.5. After repeated washing with borate buffer, the fixed stain was eluted with 0.1 N HCl/ethanol 1:1 (vol/vol). Absorbance was measured at 650 nm with the 680 Microplate Reader (Bio-Rad).

### Cubilin blocking

To see whether cubilin mediates albumin uptake, a sheep polyclonal antibody against the region between aa. 36–126 of human cubilin (R&D Systems) (Supplementary Table S1) was added to podocyte media. A titration curve was performed in order to evaluate the optimal antibody concentration to use stimulating cells with 50 µg/ml of BSA-FITC. Mature podocytes on 12-well plates were pre-incubated with 6 μg/ml of antibody for 3 h (saturation condition), then stimulated with FITC-BSA 50 µg/ml for 2 hours. This BSA concentration was used to position the experiment in the exponential phase of the uptake kinetics. The intracellular signal was measured as explained previously for the endocytotic uptake analysis. Fluorescence was normalized to the quantity of cells measured by methylene blue staining, as explained above. Results were expressed as the -fold increase versus untreated control cells.

### Statistical analysis

The statistical analysis was performed using non-parametric tests (the Mann-Whitney U test) due to the small sample size. Statistical significance was assessed using Primer software (McGraw-Hill). Results with p values below 0.05 were considered significant. Results were presented as mean ± standard deviation. For the Real Time PCR experiments, data were shown as the geometrical mean ± standard error of the mean since data were compared on three different housekeeping genes. To test Pearson’s correlation coefficient between megalin/cubilin and FITC-BSA in the colocalization experiments LAS-AF software was used. In colocalization experiments negative control was provided by quantifying Pearson’s correlation coefficient after rotation of one image by 90°.

### Data Availability Statement

The datasets generated during and/or analyzed during the current study are available from the corresponding author on reasonable request.

## Electronic supplementary material

Supplementary Information
