# Supplementary material for: Albumin uptake in human podocytes: a possible role for the cubilin-amnionless (CUBAM) complex
Source: Sci Rep. 2017 Oct 20;7:13705. doi: 10.1038/s41598-017-13789-z (PMC5651885; doi:10.1038/s41598-017-13789-z)
Supplement: Supplementary file 1 — Supplementary Information [file 41598_2017_13789_MOESM1_ESM.doc]

**Supplementary Information**

**Albumin uptake in human podocytes: a possible role for the cubilin-amnionless (CUBAM) complex**

Lisa Gianesello^1^, Giovanna Priante^1^, Monica Ceol^1^, Claudia M. Radu^2^, Moin A. Saleem^3^, Paolo Simioni^2^, Liliana Terrin^1^, Franca Anglani^1^, *Dorella Del Prete^1^

**Supplementary Figure S1. Albumin stimulation does not affect podocyte cell death or differentiation.** (A) Cell death after albumin stimulation for different times and at different doses. There were no significant differences vis-à-vis control cells, indicating that albumin did not induce podocyte death in our experimental conditions. Data are given as the average ± SD of two different experiments. (B) Expression of the podocyte-specific marker podocin after albumin stimulation for different times and at different doses. Albumin stimulation did not affect podocin expression at any of the time points analyzed. Data are given as the average ± SD of four different experiments.

**
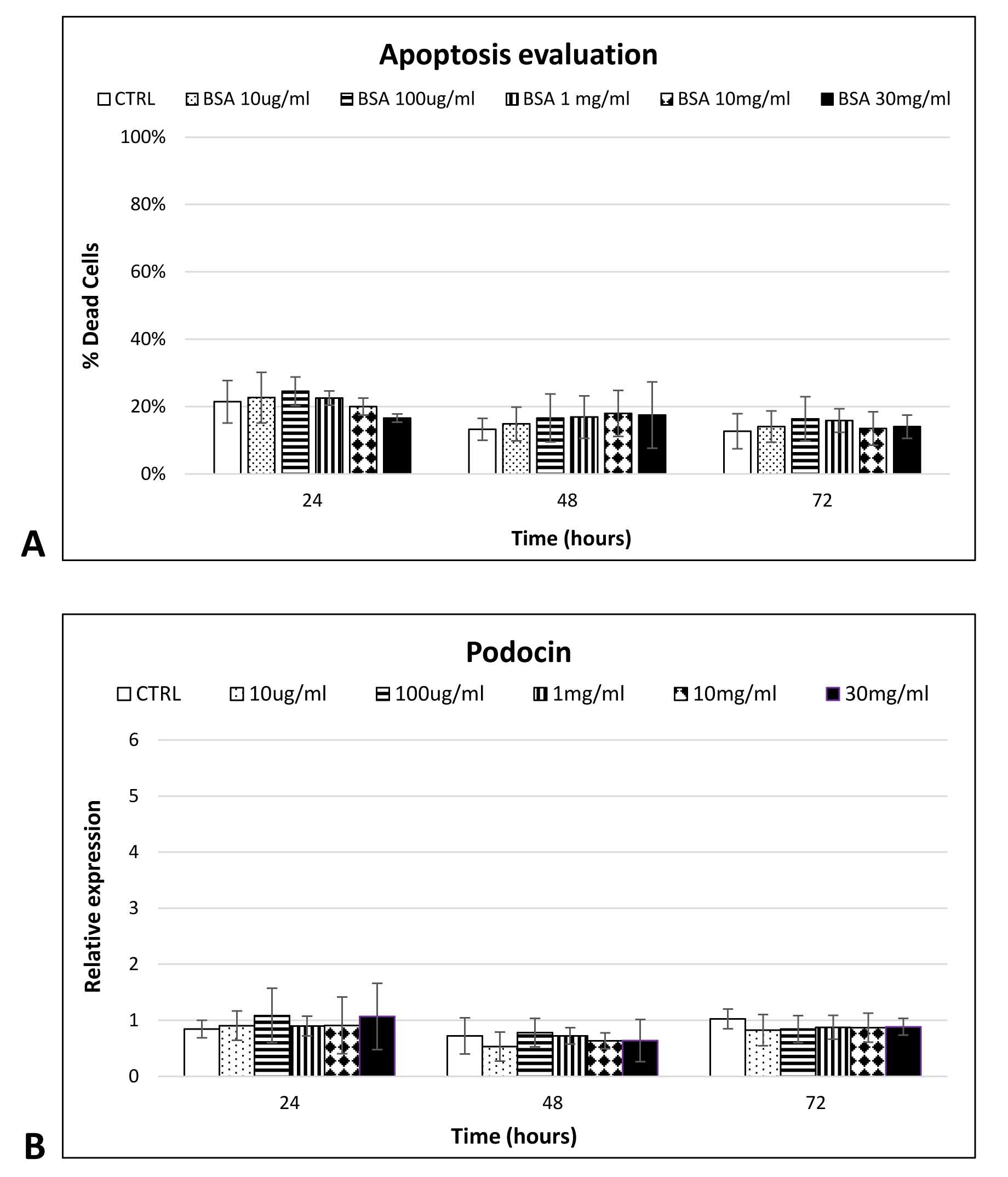
**

Supplementary Figure S1

**Table S1**: Antibodies used.

| **Target** | **Clone** | **Host** | **Manufacturer** | **Code** | **Conjugation** | **Dilution IHC/IF** | **Dilution ICW** |
| --- | --- | --- | --- | --- | --- | --- | --- |
| Podocin | H-130 | rabbit | Santa Cruz Biotechnology | sc-21009 |  | 1:50 | 1:100 |
| ClC-5 | - | rabbit | Sigma-Aldrich | HPA000401 |  | 1:200 | 1:200 |
| Megalin | - | rabbit | LS-Bio | LS-B105 |  | 1:100 | 1:100 |
| Cubilin | - | sheep | R&D Systems | AF3700 |  | 1:50 | 1:200 |
| AMN | H-281 | rabbit | Santa Cruz Biotechnology | sc-135178 |  | 1:50 | 1:100 |
| Dab2 | H-110 | rabbit | Santa Cruz Biotechnology | sc-13982 |  | 1:50 | 1:100 |
| Anti-rabbit | - | donkey | Santa Cruz Biotechnology | sc-362291 | CFL 647 | 1:100 |  |
| Anti-sheep | - | donkey | Santa Cruz Biotechnology | sc-2477 | Rhodamine | 1:100 |  |
| Anti-sheep | - | donkey | Santa Cruz Biotechnology | sc-2476 | FITC | 1:100 |  |
| Anti-rabbit | - | donkey | LI-COR | 926-32213 | IRDye 800CW |  | 1:800 |
| Anti-sheep | - | donkey | ThermoFisher  Scientific | A-21102 | Alexa Fluor 680 |  | 1:1,000 |

**Table S2**: Primer used in Real Time PCR analyses

| **Name** | **NCBI Reference Sequence** | **Sequence** |
| --- | --- | --- |
| GLIC For | NM_17851.1 | GAAGGTGAAGGTCGGAGT |
| GLIC Rev |  | TGGCAACAATATCCACTTTACCA |
| B2M For | NM_004048.2 | TCTCTCTTTCTGGCCTGGAG |
| B2M Rev |  | TCTCTGCTGGATGACGTGAG |
| HPRT1 For | NM_000194.2 | CCTGGCGTCGTGATTAGTGA |
| HPRT1 Rev |  | TCTCGAGCAAGACGTTCAGT |
| CLCN5 For | NM_000084.4 | TGCTGGAACTCTGAGCATGT |
| CLCN5 Rev |  | TACGGCAAGGAAGGCAAATA |
| CUBN For | NM_001081.3 | GCCGTGAGAAAGGATTTCAG |
| CUBN Rev |  | TCCTTGTTTGGTGGATACCTG |
| LRP2 For | NM_004525.2 | TGGGTTGACTCTCGGTTTGA |
| LRP2 Rev |  | CACGGCCATCTTTGTCCAAT |
| AMN For | NM_030943.3 | TGTCAGTCCTGGTGCAAGAA |
| AMN Rev |  | TCTGAGACGCCGAATCCG |
| Dab2 For | NM_001343.3 | GCACCAAAAGCACCCTCAAA |
| Dab2 Rev |  | CATCGCCTTTGAACCTTGCT |

**Table S3**: Real Time PCR amplification conditions

| **Name** | **[PRIMER] µM** | **T°_a_ (°C)** | **Size** | **Efficiency** |
| --- | --- | --- | --- | --- |
| GLIC For | 0.4 | 62 | 92 bp | 96% |
| GLIC Rev | 0.4 |
| B2M For | 0.4 | 62 | 72 bp | 87% |
| B2M Rev | 0.4 |
| HPRT1 For | 0.4 | 62 | 140 bp | 86% |
| HPRT1 Rev | 0.4 |
| CLCN5 For | 0.2 | 64 | 162 bp | 99% |
| CLCN5 Rev | 0.2 |
| CUBN For | 0.4 | 62 | 118 bp | 85% |
| CUBN Rev | 0.4 |
| LRP2 For | 0.4 | 64 | 156 bp | 98% |
| LRP2 Rev | 0.4 |
| AMN For | 0.1 | 62 | 100 bp | 96% |
| AMN Rev | 0.4 |
| Dab2 For | 0.4 | 62 | 85 bp | 97% |
| Dab2 Rev | 0.2 |
